# Supplementary material for: Associations Between Care Environments and Environmental Modifications in the Daily Living Settings of Children with Medical Complexity
Source: Nurs Rep. 2025 Nov 13;15(11):400. doi: 10.3390/nursrep15110400 (PMC12655564; doi:10.3390/nursrep15110400)
Supplement: Supplementary file 1 [file nursrep-15-00400-s001.zip › Table S1. Items Related to the Care Environment.pdf]

Table S1. Items Related to the Care Environment

**Physical Environment**
An environment that is comfortable and suited to the child's physical functions
An environment that ensures the child's safety
An environment free from infection risk
An environment that facilitates childcare and nurturing
An environment with reliable access to electricity and communication
An environment that allows the child go out easily
An environment that enables the family go out easily
**Collaborative Environment**
An environment that promotes the healthy growth and development of the child
An environment that enables the child to engage in activities easily
An environment that enables the family to participate in activities and take on roles
An environment that supports the child's decision-making
An environment that supports family decision-making
An environment that enables the family to acquire care skills
An environment that affirms family participation in care and child-rearing
An environment that enables the family to provide necessary care for the child
An environment where family members cooperate
An environment that enables the family to rebuild failing relationships
An environment that allows the family to rest
An environment that allows the family to live at their own pace
An environment that promotes communication between the child, family, and professionals
An environment where the child and family have reliable professionals
**Service Environment**
An environment with medical backup to support the child's care
An environment where professionals respect the wishes and intentions of the child and family
An environment where professionals meet the needs of the family
An environment where professionals consider the child's background and condition

An environment where the child and family can receive necessary financial support
An environment where the child and family can access needed services
An environment that fosters good relationships among professionals
An environment where professionals strive to maintain and improve the quality of home care
An environment where the family can easily access a contact point
An environment where service coordination is provided when needed
**Community Environment**
An environment that guarantees employment for family members
An environment where the child and family feel accepted
An environment where the child and family connect with community members
An environment where necessary services are provided within the community
