# Supplementary material for: Associations Between Care Environments and Environmental Modifications in the Daily Living Settings of Children with Medical Complexity
Source: Nurs Rep. 2025 Nov 13;15(11):400. doi: 10.3390/nursrep15110400 (PMC12655564; doi:10.3390/nursrep15110400)
Supplement: Supplementary file 1 [file nursrep-15-00400-s001.zip › Table S2. Items Related to Environmental Modifications.pdf]

**Table S2. Items Related to Environmental Modifications**

| **Physical modifications** | **Agent** |
|---|---|
| Families procured supplies necessary for the child’s care and upbringing | F |
| Families structured the environment to promote the child’s growth and development | F |
| Families and professionals confirmed with each other the preparations needed to provide care for the child in daily living settings | F&P |
| Families and professionals collaborated to adjust the environment to promote the child’s physical and mental growth and development | F&P |
| Professionals supported families in maintaining their living space | P |
| Professionals supported families in preparing for emergencies and disasters | P |
| **Family-led environmental modifications** | |
| Families made decisions regarding the child’s medical care and upbringing | F |
| Families cooperated so that all family members could be involved with the child | F |
| Families acquired medical care and childrearing skills at their own pace | F |
| Families adjusted the timing of the child’s care while considering the overall life of the family | F |
| Families made efforts to manage their own physical and mental health | F |
| Families sought to enhance their motivation for childrearing | F |
| Families were aware of their social roles | F |
| **Family-led, facilitated by the professional role, environmental modifications** | |
| Families consulted professionals about concerns and difficulties related to the child’s health and upbringing | F |
| Families asked professionals about things they did not understand based on the information provided | F |
| Families communicated their thoughts about the child and their views on childrearing to professionals | F |
| Families gathered information on local services and social resources | F |
| Families obtained information about the necessary financial support | F |
| Families prepared for responses to emergencies and disasters | F |
| Families reported to professionals about changes resulting from service use | F |
| Families and professionals discussed the kind of life and childrearing the family desired | F&P |
| Families and professionals discussed childrearing approaches that made use of the family’s life circumstances and background | F&P |
| Families and professionals discussed the healthy growth and development of siblings | F&P |
| Families and professionals collaborated in providing care for the child | F&P |
| Families and professionals discussed challenges in childrearing | F&P |
| Professionals supported the decision-making of the child and family | P |
| **Community environmental modifications** | |
| Families advocated for making the community more livable for the child and family | F |
| Families accepted the child’s connection with the community | F |

| | |
|---|---|
| Professionals supported the child and family in building connections with the community | P |
| Professionals engaged with the community to make it more accommodating for the child and family | P |
| **Service environmental modifications** | |
| Families utilized services | F |
| Families and professionals collaborated to develop comprehensive care plans for community living | F&P |
| Professionals supported families in achieving their desired lifestyle and childrearing goals | P |
| Professionals respected family life in the course of providing support | P |
| Professionals supported families in selecting necessary services | P |
| Professionals provided information to reduce families' financial burden | P |
| Professionals collaborated with other relevant disciplines involved in the child and family's care | P |
| Professionals supported families in assessing the child's condition | P |
| Professionals supported families in maintaining and promoting their health | P |
| Professionals developed systems for providing necessary community services | P |
| Professionals continued learning to improve the quality of support for children requiring medical care | P |
| **Care improvement environmental modifications** | |
| Families and professionals discussed challenges related to services | F&P |
| Professionals provided support with consideration for the family's acceptance process, physical and mental condition, and relationships | P |
| Professionals provided information using terminology that was easy for families to understand | P |
| Professionals actively addressed families' concerns and difficulties | P |
| Professionals supported families in working together to provide childrearing and care | P |
| Professionals provided guidance on medical care and childrearing tailored to the family | P |
| Professionals supported families in adjusting the child's living environment | P |

Note. Agents of environmental modifications: Family-led (F); Family and professional jointly (F&P); Professional-led (P).
