# Supplementary material for: Associations Between Care Environments and Environmental Modifications in the Daily Living Settings of Children with Medical Complexity
Source: Nurs Rep. 2025 Nov 13;15(11):400. doi: 10.3390/nursrep15110400 (PMC12655564; doi:10.3390/nursrep15110400)
Supplement: Supplementary file 1 [file nursrep-15-00400-s001.zip › Table S3._Participant Characteristics.pdf]

Table S3. Participant Characteristics

Family characteristics N=90

Item		N（%）
Primary caregiver (multiple responses)）	Mother	87(96.7%)
	Father	12(13.3%)
	Grandparent(s)	4(4.4%)
	Other	1(1.1%)
Presence of siblings	Yes	65(72.2%)
	No	25(27.8%)
Number of siblings (n = 65)	1	43(47.8%）
	2	12(13.3%)
	3 or more	10(1.1%)
Employment status of primary caregiver	Yes	44(48.9%)
	No	46(51.1%)
Interaction with extended family	Always/Sometimes	58(64.4%)
	Rarely/Never	32(35.6%)
Presence of a person to consult with	Yes	76(84.4%)
	No	14(15.6%)
Family relationships (n = 89)	Good/Somewhat good	85(95.5%)
	Somewhat poor/Poor	4(4.5%)

Professional characteristics N＝221

Item		N（%）
Occupation at the time of involvement in environmental modifications	Visiting nurse	141(63.8%)
	Consultation Support Specialist	34(15.4%)
	nurses working at child development support centers and after-school day service centers	27(12.2%)
	Hospital nurse	10(4.5%)
	Medical care coordinator for children	9(4.1%)
Presence of other institutions or professionals available for consultation	Yes	184(83.3%）
	No	37(16.7%)
Relationships among professionals （n=219)	Good/Somewhat good	205(93.6%)
	Somewhat poor/Poor	14(6.4%)
Experience with service coordination （n=220)	Yes	106(48.2%)
	No	114(51.8%)
Participation in training sessions or study groups	Yes	178(80.5%)
	No	43(19.5%)

**Environmental modification case characteristics**		N = 311
Item		N（%）
Primary diseases of CMC	Neurological, gastrointestinal, and respiratory disorders	211(67.8%)
	Neurological and gastrointestinal disorders	13(4.2%)
	Neurological, gastrointestinal, and excretory disorders	12(3.9%)
	Neurological and respiratory disorders	8(2.6%)
	Neurological, gastrointestinal, excretory, and respiratory disorders	1(0.3%)
	Gastrointestinal and respiratory disorders	30(9.6%)
	Gastrointestinal disorders	11(3.5%)
	Gastrointestinal and excretory disorders	5 (1.6%)
	Respiratory disorders	11(3.5%)
	Excretory disorders	5(1.6%)
	Excretory and respiratory disorders	3(1.0%)
	Others	1(0.3%)
Context of environmental modification	Preparation for transition to daily living	145(46.6%)
	Change in CMC care methods	51(16.4%)
	Change in CMC health condition	44(14.1%)
	CMC life events	38(12.2%)
	Family health problems	9(2.9%)
	Family life events	7(2.3%)
	Sibling life events	5(1.6%)
	Other	12(3.9%)
Child’s health status (n=310)	Stable	148(47.7%)
	Unstable	162(52.3%)
Child’s signs and responses (n=310)	Readable	82(26.5%)
	Difficult to read	228(73.5%)
Child’s expression of intent	Understandable	86(27.7%)
	Not understandable	225(72.3%)
Desired services	Present	163(52.8%)

(n=309)	Absent	92(29.8%)
	Not applicable to services	54(17.5%)
Service accessibility (n=304)	Easy to use	123(40.5%)
	Difficult to use	121(39.8%)
	Not applicable to services	60(19.7%)
Information about necessary services (n=310)	Sufficient/Some	196（63.2%）
	Little/None	114（36.8%）
People the family communicates with	Present	217(69.8%)
	Absent	49(15.8%)
	Unknown	45（14.5%）
People the family can ask for help	Present	172(55.3%)
	Absent	82(26.4%)
	Unknown	57（18.3%）
People the family can share private matters with	Present	161(51.8%)
	Absent	60(19.3%)
	Unknown	90（28.9%）
