# Supplementary material for: Associations Between Care Environments and Environmental Modifications in the Daily Living Settings of Children with Medical Complexity
Source: Nurs Rep. 2025 Nov 13;15(11):400. doi: 10.3390/nursrep15110400 (PMC12655564; doi:10.3390/nursrep15110400)
Supplement: Supplementary file 1 [file nursrep-15-00400-s001.zip › Table S4._Differences in Environmental Modification Scores between Families and Professionals, and by Agent of Modification.pdf]

Table S4. Differences in Environmental Modification Scores between Families and Professionals, and by Agent of Modification

		**Group 1: families** Median(IQR,N)	**Group2: professionals** Median(IQR,N)	U	Z value	*p* value	Effect size (*r*)
By type of environmental modification	Total environmental modifications score	173(194-151,45)	185(200-166,100)	2734	2.067	0.039*	0.172
	Total physical environmental modifications score	22(24-18,74)	23(26-21,195)	8721	2.651	0.008**	0.162
	Total family-led environmental modifications score	25(28-20,64)	26(30-23,144)	5378	1.926	0.054	0.134
	Total Family-led, facilitated by the professional role, environmental modifications score	47(53-40,58)	51(55-44,128)	4616	2.660	0.008**	0.195
	Total community environmental modifications score	11(14-8,74)	12(15-10,163)	7047	2.084	0.037*	0.135
	Total service environmental modifications score	39(44-32,61)	42(46-37,177)	6860	3.154	0.002**	0.204
	Total care improvement environmental modifications score	27(29-21,75)	28(32-26,201)	9559	3.440	<0.001**	0.207
By agent	Family-led environmental modifications	68(77-58,62)	70(79-60,128)	4358	1.098	0.272	0.080
	Family–professional joint environmental modifications	30(35-24,62)	35(39-31,160)	6865	4.443	<0.001**	0.298
	Professional-led environmental modifications	69(80-58,60)	76(83-68,174)	6568	2.983	0.003**	0.195

Note. Results are based on Mann–Whitney U tests; $^{**}p < 0.01$, $^{*}p < 0.05$.

Effect size (r) was calculated as $r = |Z| / \sqrt{N}$, where N is the total sample size of the two groups combined.

Total sample = 311, including 90 families and 221 professionals.
