# Supplementary material for: Associations Between Care Environments and Environmental Modifications in the Daily Living Settings of Children with Medical Complexity
Source: Nurs Rep. 2025 Nov 13;15(11):400. doi: 10.3390/nursrep15110400 (PMC12655564; doi:10.3390/nursrep15110400)
Supplement: Supplementary file 1 [file nursrep-15-00400-s001.zip › Table S5._Differences in Care Environment Scores before and after Environmental Modifications.pdf]

Table S5. Differences in Care Environment Scores Pre- and Post-Environmental Modifications

Total sample=311

	**Environment [pre]** Median (IQR)	**Environment [post]** Median (IQR)	Z value	*p* value	Effect size (*r*)
Total care environment score N=199	116[137-101]	152[168-134]	-11.757	$p < 0.001$**	0.833
Total physical environment score N=272	25[30-18]	32[35-29]	-12.702	$p <0.001$**	0.770
Total collaborative environment score N=229	47[56-39]	59[69-53]	-12.191	$p < 0.001$**	0.806
Total service environment score N=250	32[37-26]	44[50-38]	-11.698	$p < 0.001$**	0.740
Total community environment score N=261	12[14-9]	16[18-12]	-13.422	$p < 0.001$**	0.831

Note. Results are based on the Wilcoxon signed-rank test; ** $p < 0.01$.

Effect size (r) was calculated as r=|Z|/√N, where Z is the standardized test statistic and N is the number of pairs used in the analysis.
