# Supplementary material for: Associations Between Care Environments and Environmental Modifications in the Daily Living Settings of Children with Medical Complexity
Source: Nurs Rep. 2025 Nov 13;15(11):400. doi: 10.3390/nursrep15110400 (PMC12655564; doi:10.3390/nursrep15110400)
Supplement: Supplementary file 1 [file nursrep-15-00400-s001.zip › Table S6._Associations between Environmental Modifications and the Post-modification Care Environment.pdf]

TableS6. Associations between Environmental Modifications and the Post-modification Care Environment

Total sample=311

Dependent variable: Post-modification care environment	Post-modification N=275 Physical environment		Post-modification N=233 Collaborative environment		Post-modification N=262 Community environment		Post-modification N=254 Service environment	
	**Standardized coefficient *β***	***P* value**	**Standardized coefficient *β***	***P* value**	**Standardized coefficient *β***	***P* value**	**Standardized coefficient *β***	***P* value**
Independent variable: Environmental modification								
● Physical modifications	0.320	0.052	0.396	0.025**	0.042	0.799	0.137	0.404
● Family-led environmental modifications	0.216	0.061	0.331	0.006**	0.212	0.058	0.305	0.006**
● Family-led, facilitated by the professional role, environmental modifications	-0.067	0.653	-0.148	0.327	-0.229	0.114	-0.288	0.037
● Community environmental modifications	-0.191	0.291	-0.074	0.680	-0.250	0.147	-0.192	0.262
● Service environmental modifications	0.027	0.805	0.113	0.313	0.176	0.112	-0.024	0.827
● Care improvement environmental modifications	0.219	0.245	0.029	0.885	0.603	0.001**	0.686	<0.001**
Adjusted R^2	0.194		0.306		0.247		0.311	

Note. Results are based on multiple regression analysis; $^{**}p<0.01$, $^{*}p<0.05$.
